# Vitamin D Modulates Hematological Parameters and Cell Migration into Peritoneal and Pulmonary Cavities in Alloxan-Diabetic Mice

**DOI:** 10.1155/2017/7651815

**Published:** 2017-04-19

**Authors:** Leonardo M. Bella, Isis Fieri, Fernando H. G. Tessaro, Eduardo L. Nolasco, Fernanda P. B. Nunes, Sabrina S. Ferreira, Carolina B. Azevedo, Joilson O. Martins

**Affiliations:** ^1^Laboratory of Immunoendocrinology, Department of Clinical and Toxicological Analyses, Faculty of Pharmaceutical Sciences, University of Sao Paulo (FCF/USP), São Paulo, SP, Brazil; ^2^Department of Medicine, Rheumatology Division, Universidade Federal de São Paulo, São Paulo, SP, Brazil

## Abstract

*Background/Aims*. The effects of cholecalciferol supplementation on the course of diabetes in humans and animals need to be better understood. Therefore, this study investigated the effect of short-term cholecalciferol supplementation on biochemical and hematological parameters in mice.* Methods*. Male diabetic (alloxan, 60 mg/kg i.v., 10 days) and nondiabetic mice were supplemented with cholecalciferol for seven days. The following parameters were determined: serum levels of 25-hydroxyvitamin D, phosphorus, calcium, urea, creatinine, alanine aminotransferase, aspartate aminotransferase, alkaline phosphatase, red blood cell count, white blood cell count (WBC), hematocrit, hemoglobin, differential cell counts of peritoneal lavage (PeL), and bronchoalveolar lavage (BAL) fluids and morphological analysis of lung, kidney, and liver tissues.* Results*. Relative to controls, cholecalciferol supplementation increased serum levels of 25-hydroxyvitamin D, calcium, hemoglobin, hematocrit, and red blood cell counts and decreased leukocyte cell counts of PeL and BAL fluids in diabetic mice. Diabetic mice that were not treated with cholecalciferol had lower serum calcium and albumin levels and hemoglobin, WBC, and mononuclear blood cell counts and higher serum creatinine and urea levels than controls.* Conclusion*. Our results suggest that cholecalciferol supplementation improves the hematological parameters and reduces leukocyte migration into the PeL and BAL lavage of diabetic mice.

## 1. Introduction

Type 1 diabetes mellitus (T1D) is an autoimmune disorder that causes destruction of pancreatic *β* cells and insulitis resulting in loss of insulin secretion [1].

Several studies have demonstrated that diabetic individuals with uncontrolled hyperglycemia may experience weight loss, uncontrolled immune response, hematological alterations, and lung, liver, and kidney injuries [2, 3]. In addition, renal impairment in diabetes may affect the metabolism of urea, creatinine [4], hemoglobin (Hb) [5], albumin [6], and vitamin D [7, 8].

Anemia is defined as a decrease in the amount of hemoglobin (Hb) or the number of red blood cells (RBC) in the blood. It is a common complication in patients with diabetic kidney disease and it increases mortality in diabetic individuals, but its mechanism of action is still not clear [9].

Albumin is the major plasma protein and plays a critical role in maintaining tissue oncotic pressure and in the transport of substances such as vitamin D into the bloodstream. Studies have suggested that hepatic albumin synthesis and secretion are reduced in diabetic rats [10].

Vitamin D is a hormone obtained from the diet and/or by endogenous synthesis. The vitamin D molecule is hydroxylated twice, once in the liver by the enzyme 25–hydroxylase and once in the kidney by the enzyme 1*α*–hydroxylase, to form calcitriol (1,25(OH)_2_D), which is the form of vitamin D that interacts with vitamin D receptor (VDR) to perform its biological activity [7].

The classic activity of calcitriol is to regulate bone metabolism by maintaining a balance between calcium (Ca) and phosphorus (P) concentrations [8]. Studies have reported that vitamin D may affect the course of T1D by immunomodulation [11]. Although the mechanism of action is still unknown, serum 1,25(OH)_2_D levels appear to modulate the level of systemic cytokine production and to increase white blood cell (WBC) count [12, 13].

Although vitamin D supplementation is an important aid in clinical practice, few studies have demonstrated the effects of vitamin D supplementation on the course of T1D in humans and animals. In addition, vitamin D supplementation studies are often contradictory due to the lack of precise information on regimen and dosage protocols [14].

Thus, using a well-established mouse model of T1D, we investigated the effect of vitamin D supplementation on histological, biochemical, and hematological parameters and on bronchoalveolar lavage (BAL) and peritoneal lavage (PeL) fluids in male (C57BL/6) diabetic and nondiabetic mice. The hypothesis is that vitamin D supplementation improves the hematological parameters and reduces inflammatory cell migration into the PeL and BAL fluid of diabetic mice.

## 2. Materials and Methods

### 2.1. Animals

Thirty-one specific pathogen-free male C57BL/6 mice weighing 25 ± 2 g at baseline were used. The animals were maintained at 22°C under a 12 h light-dark cycle. Food and water were provided ad libitum before and during the experimental period. This study was conducted in strict accordance with the principles and guidelines of the National Council for the Control of Animal Experimentation (CONCEA) and approved by the Ethics Committee on Animal Use (CEUA) at the School of Pharmaceutical Sciences (FCF), University of São Paulo, Brazil (protocol number: CEUA/FCF/389). Surgery was performed under ketamine/xylazine anesthesia and all efforts were made to minimize animal suffering.

### 2.2. Induction of Diabetes Mellitus

Animals were separated in four groups: control (C), control supplemented with vitamin D (CV), diabetic (D), and diabetic supplemented with vitamin D (DV). Briefly, diabetes mellitus was induced by intravenous injection of 60 mg/kg alloxan monohydrate (ALX) (Sigma Chemical Co., St. Louis, MO, USA) dissolved in physiological saline (0.9% NaCl). Control mice were injected with physiological saline only. Ten days after diabetes induction, body weight was measured and peripheral blood (tail vein) glucose level was determined using an Accu-Chek Advantage II blood glucose monitor (Roche Diagnóstica, São Paulo, SP, Brazil). Animals were considered diabetic if glucose level was above 300 mg/dL [15].

### 2.3. Vitamin D Supplementation

Animals in the CV and DV groups were supplemented orally with 800 IU of vitamin D (Sanofi Aventis, São Paulo, SP, Brazil) for seven days [14]. The last dose of vitamin D was administered 24 h before the experiment to minimize stress-induced endocrine changes caused by oral administration of the hormone [16]. Body weight and blood glucose were measured on the first, fourth, and seventh day of the supplementation period.

### 2.4. Blood Collection

Animals were anesthetized by an intraperitoneal injection of 10 mg/kg xylazine hydrochloride (Ceva Santé Animale, Paulínia, SP, Brazil) and 90 mg/kg ketamine hydrochloride (Ceva Santé Animale). Blood was collected from anesthetized animals by cardiac puncture. Samples of EDTA-anticoagulated blood were used to determine the following hematological parameters: red blood cell (RBC) count, white blood cell (WBC) count, hematocrit (Htc), and hemoglobin (Hb). All analyses were performed using a veterinary hematology analyser ABC Vet (HORIBA®, UK). Differential cell counts were determined on stained slides under oil immersion microscopy. Based on morphological criteria, 100 cells per sample were counted and classified as either mononuclear or polymorphonuclear. A sample aliquot without anticoagulant was centrifuged (10 min, 3500 rpm) at room temperature. Next, the serum was separated and used for measuring ionic calcium (Ca), phosphorus (P), albumin, alanine transaminase (ALT), aspartate aminotransferase (AST), alkaline phosphatase (ALP), urea, and creatinine by colorimetric assay according to the manufacturer's protocol (LabTest Diagnóstica, Lagoa Santa, MG, Brazil). Serum 25(OH)D was measured by EUROIMMUN 25–OH–Vitamin D ELISA kit (Luebeck, Germany). Serum samples were stored at −80°C.

### 2.5. Peritoneal and Bronchoalveolar Lavage

The abdomen of all mice was exposed through a ventral midline incision. Peritoneal lavage (PeL) was performed by instillation of 10 mL (two 5 mL infusions) of phosphate buffered saline (PBS; 137 mM NaCl, 2.7 mM KCl, 4.3 mM Na_2_HPO_4_, and 1.47 mM KH_2_PO_4_; pH 7.4) at room temperature [17]. The trachea was exposed through a midline ventral incision in the neck. Bronchoalveolar lavage (BAL) was performed by instillation of 5 mL (five 1 mL infusions) of PBS at room temperature through a 16 G × 1.88′′ BD Angiocath™ polyethylene tube (BD, Franklin Lakes, NJ, USA) inserted into the trachea. Next, PeL and BAL samples were centrifuged (1500 rpm, 10 min, 4°C), supernatants were discarded, and the cells were resuspended in PBS (1 mL). The cell suspension was diluted 1 : 2 (v : v) with Turk's solution. Total cell counts were determined using a Neubauer chamber and differential cell counts were examined on stained slides under oil immersion microscopy. A total of 100 cells were counted and classified as neutrophils, lymphocytes, or macrophages based on morphological criteria.

### 2.6. Tissue Extraction and Histological Analysis

Kidney, liver, and lung samples were extracted and subsequently fixed in formaldehyde solution (10%). After fixation, tissues were dehydrated in increasing ethanol concentrations (70–100%), diaphonized in xylol, and embedded in paraffin. Transverse sections (5 *μ*m) obtained after inclusion were stained with hematoxylin and eosin (H/E). After staining, the material was dehydrated, diaphonized, and mounted with Entellan®. Slides containing the tissue were observed under a light microscope (Nikon Eclipse 80i, Tokyo, Japan) and photographed using the NIS-Elements AR imaging software (Nikon).

### 2.7. Data and Statistical Analysis

The data were analysed by analysis of variance (ANOVA) followed by the Tukey-Kramer multiple comparisons test (when appropriate) or unpaired *t*-test using GraphPad Prism 6.0 software (La Jolla, CA, USA). A two-tailed *p* value with 95% confidence interval was computed. Data are presented as mean ± standard deviation (SD). Data were considered significant when *p* < 0.05.

## 3. Results

### 3.1. Body Weight Gain and Blood Glucose Levels

Ten days after administration of ALX, diabetic mice exhibited sharply elevated blood glucose (mean ± SD; control, 165.6 ± 5.7 mg/dL, *n* = 18; diabetic, 568.1 ± 10.0 mg/dL, *n* = 13; *p* < 0.001) levels and a reduction in body weight gain (control, 0.8 ± 0.3 g, *n* = 18; diabetic, −1.3 ± 0.4 g, *n* = 13; *p* < 0.05) compared to controls.

### 3.2. Analysis of Vitamin D Supplementation

**Figure 1:**
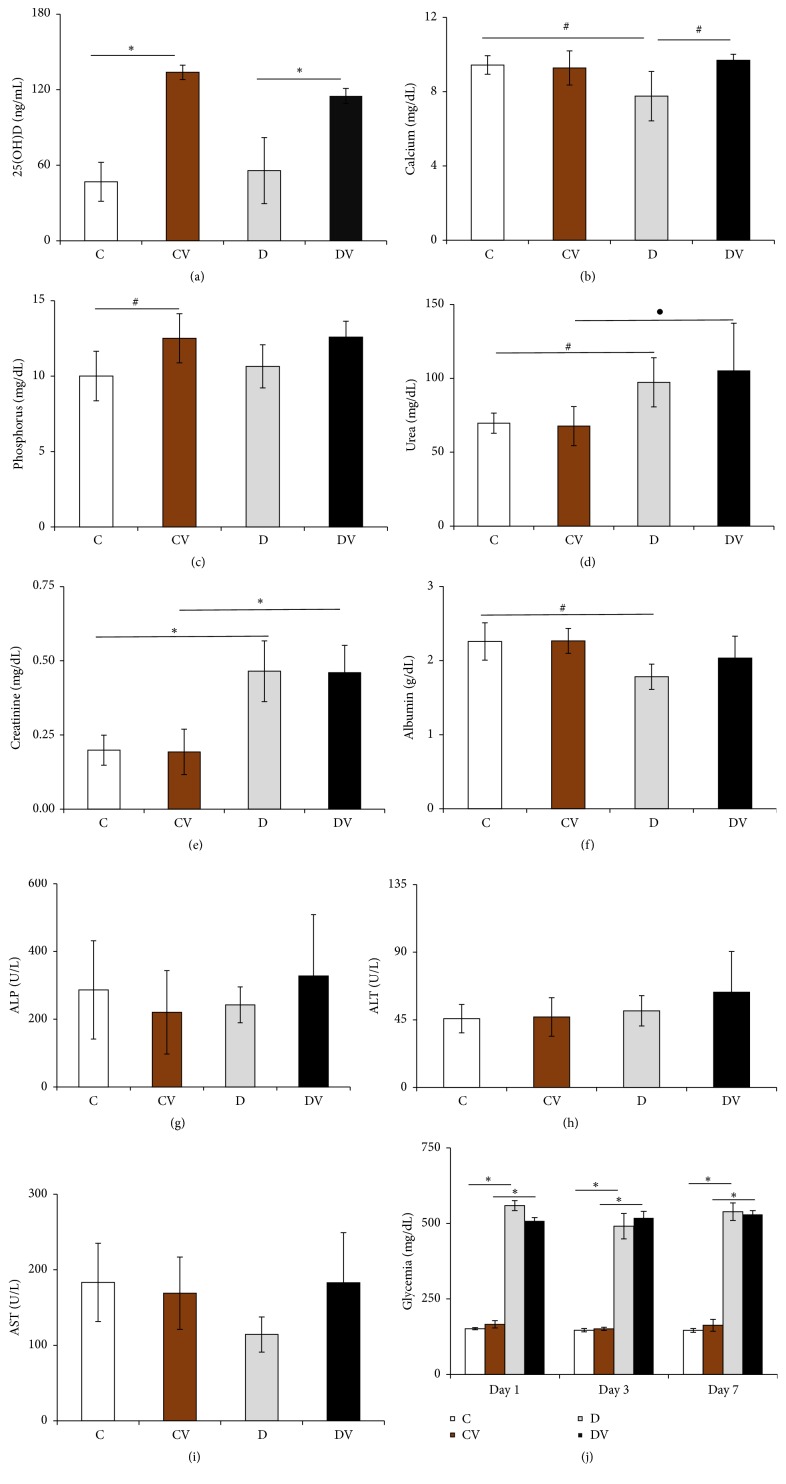
Biochemical and vitamin D levels. Data are expressed as mean and standard deviation for 3–9 animals per group. ^#^*p* < 0.05; ^●^*p* < 0,01; and ^*∗*^*p* > 0.001. 25(OH)D: 25-hydroxyvitamin D; ALP: alkaline phosphatase; ALT: alanine transaminase; AST: aspartate aminotransferase; C: control; CV: vitamin D; D: diabetic; DV: diabetic + vitamin D.

Animals supplemented with cholecalciferol (CV and DV groups) exhibited higher serum 25(OH)_2_D (ng/mL) levels than their respective controls (C and D groups). These results indicate that vitamin D supplementation was successful (Figure 1(a)).

Diabetic mice had lower serum Ca (mg/dL) levels than nondiabetic animals. However, vitamin D supplementation restored serum Ca levels in the DV group (Figure 1(b)).

Serum phosphorus (mg/dL) levels were significantly higher in vitamin D-treated nondiabetic mice compared to nondiabetic controls (Figure 1(c)).

### 3.3. Temporal Variation of Blood Glucose Levels and Body Weight

Body weight gain and blood glucose levels were measured during the supplementation period. Body weight was measured in the first and seventh days of the experiment. There was no significant difference in body weight variation between vitamin D_3_-supplemented animals (CV, 0.1 ± 0.3 g, *n* = 9; DV, −1.3 ± 1.0 g, *n* = 7) and their respective controls (C, 0.3 ± 0.3 g, *n* = 9; D, −1.1 ± 0.9 g, *n* = 6). Blood glucose was measured on the first, fourth, and seventh day of the experimental period (Figure 1(j)). There was no significant difference in glycemic evolution between vitamin D_3_-supplemented (CV and DV) groups and controls (C and D groups). However, blood glucose levels remained significantly higher in diabetic mice (D and DV groups) than in controls (C and CV).

### 3.4. Vitamin D_3_ Supplementation Improved Hematological Parameters

**Figure 2:**
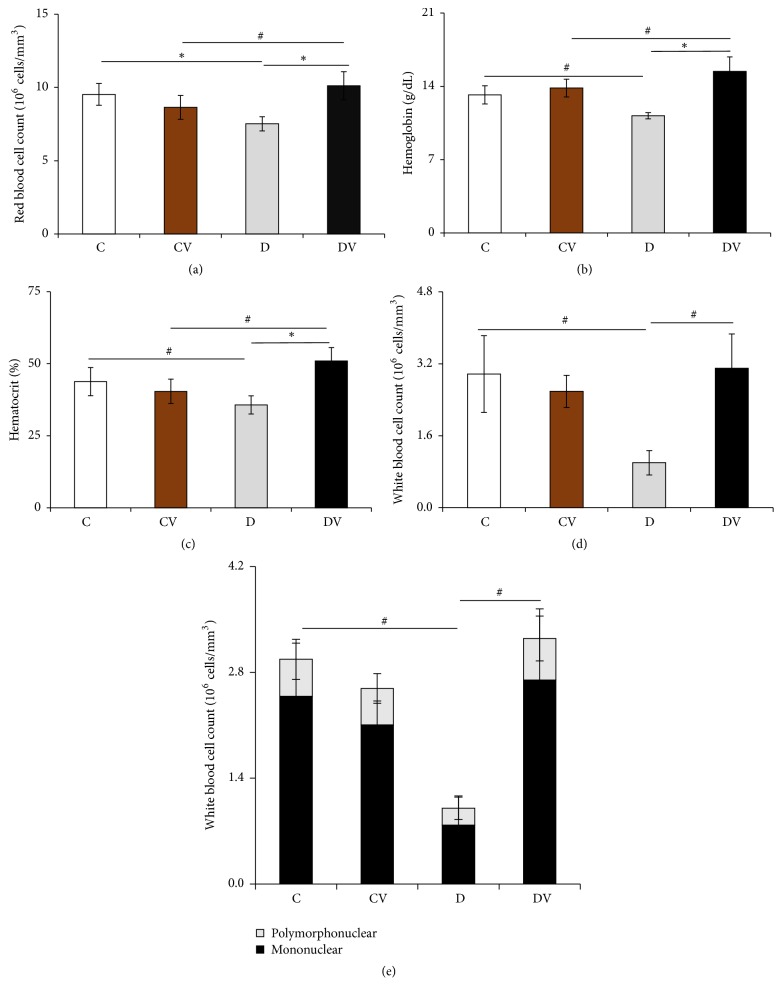
Hematological parameters. Data are expressed as mean and standard deviation for 3–9 animals per group. ^#^*p* < 0.05 and ^*∗*^*p* > 0.001. C: control; CV: vitamin D; D: diabetic; DV: diabetic + vitamin D.

Diabetic mice had lower RBC, Hb, Htc, WBC, and peripheral blood mononuclear cell counts than controls (Figure 2). Diabetic mice supplemented with vitamin D_3_ had higher RBC, Hb, Htc, WBC, and mononuclear cell counts than diabetic mice. In addition, diabetic mice supplemented with vitamin D_3_ had higher Hb and hematocrit levels than nondiabetic mice supplemented with vitamin D_3_ (Figure 2).

### 3.5. Vitamin D_3_ Supplementation in the Kidneys and Liver

**Figure 3:**
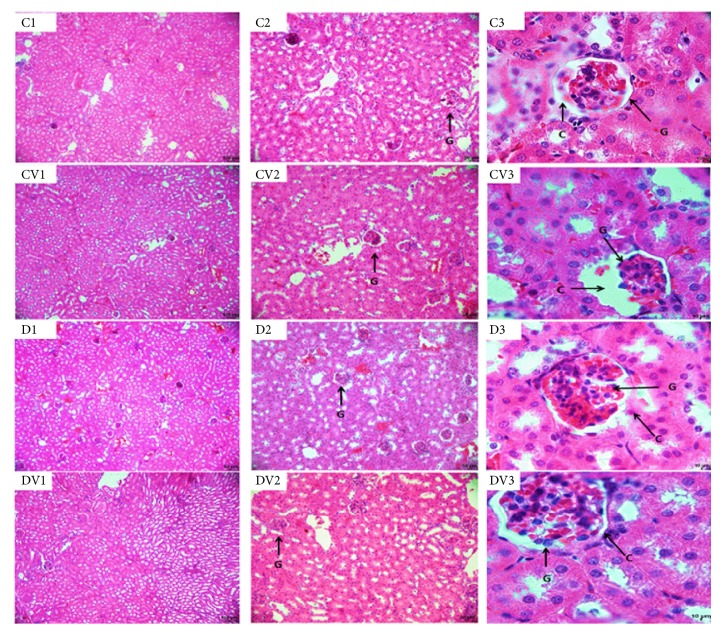
Renal tissue of mice. Photomicrograph of the mice kidney tissue. C1, C2, and C3 renal tissue of animal control; CV1, CV2, and CV3 renal tissue of animal supplemented with vitamin D; D1, D2, and D3 renal tissue of diabetic animal; DV1, DV2, and DV3 renal tissue of diabetic animal supplemented with vitamin D. Diabetic animals presented thickening of Bowman's capsule (C). Presence of glomerulus (G) (bars, C1, CV1, D1, and DV1 = 100 *μ*m; C2, CV2, D2, and DV2 = 50 *μ*m; C3, CV3, D3, and DV3 = 10 *μ*m). Staining: H/E.

**Figure 4:**
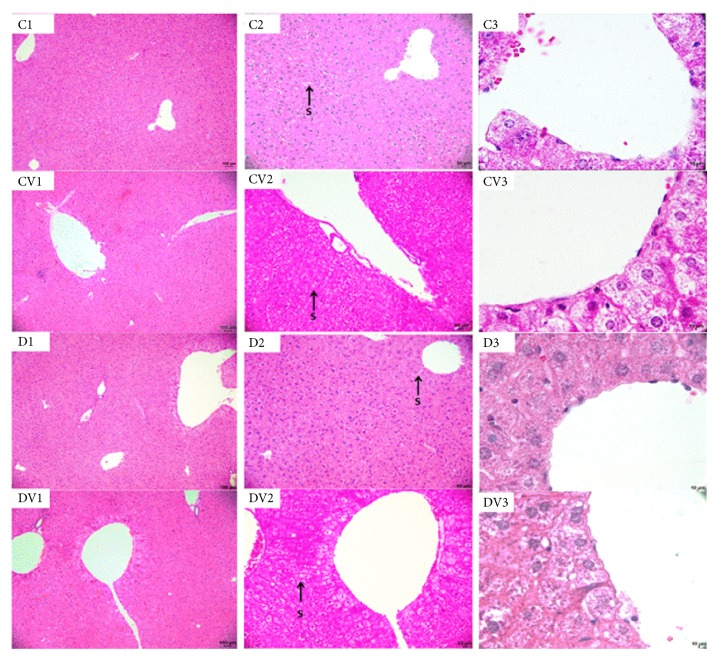
Liver tissue of mice. Photomicrograph of the mice liver tissue. C1, C2, and C3 liver tissue of animal control; CV1, CV2, and CV3 liver tissue of animal supplemented with vitamin D; D1, D2, and D3 liver tissue of diabetic animal; DV1, DV2, and DV3 liver tissue of diabetic animal supplemented with vitamin D. Liver sinusoids (S). (Bars, C1, CV1, D1, and DV1 = 100 *μ*m; C2, CV2, D2, and DV2 = 50 *μ*m; C3, CV3, D3, and DV3 = 10 *μ*m). Staining: H/E.

**Figure 5:**
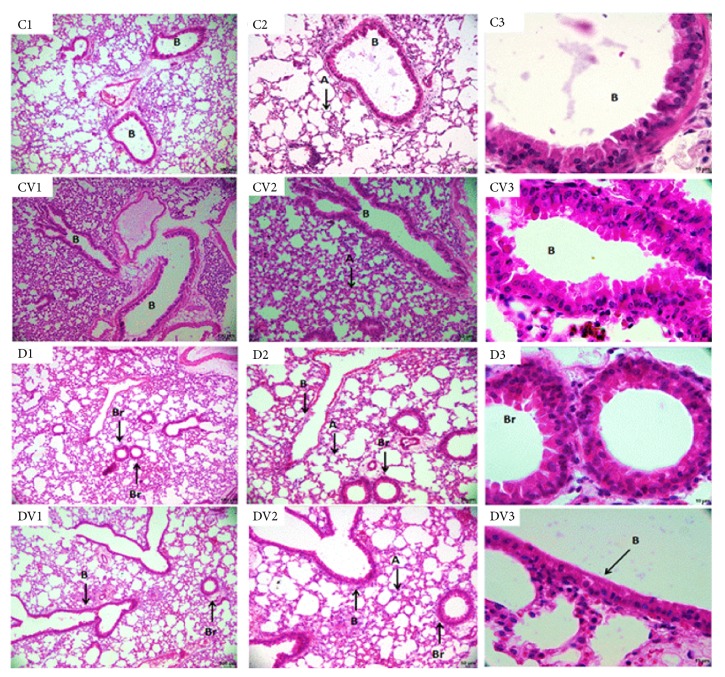
Lung tissue of mice. Photomicrograph of the mice lung tissue. C1, C2, and C3 lung tissue of animal control; CV1, CV2, and CV3 lung tissue of animal supplemented with vitamin D; D1, D2, and D3 lung tissue of diabetic animal; DV1, DV2, and DV3 lung tissue of diabetic animal supplemented with vitamin D. Lung alveolus (A); bronchus (B); bronchiole (Br). (Bars, C1, CV1, D1, and DV1 = 100 *μ*m; C2, CV2, D2, and DV2 = 50 *μ*m; C3, CV3, D3, and DV3 = 10 *μ*m). Staining: H/E.

Diabetic mice had significantly higher serum levels of urea (Figure 1(d)) and creatinine than controls (Figure 1(e)). In addition, diabetic mice had a significant reduction in serum albumin concentration compared to controls (Figure 1(f)). However, there were no significant differences in serum levels of ALP, AST, and ALT (Figures 1(g)–1(i)) between vitamin D_3_-treated mice and their respective controls. Compared to controls, diabetic mice exhibited a thickening of the Bowman's capsule. However, vitamin D supplementation did not restore this renal parameter (Figure 3). No significant differences in liver (Figure 4) and lung (Figure 5) morphology were observed between vitamin D_3_-supplemented animals (CV and DV) and their respective controls.

### 3.6. Cell Composition of Bronchoalveolar Lavage and Peritoneal Lavage Fluids

The PeL fluid of vitamin D_3_-supplemented mice had 4.9-fold more neutrophils than respective controls, but no significant difference in total cell counts was observed across groups. Total leukocyte counts in the PeL fluid were reduced by 47% in vitamin D_3_-treated diabetic mice compared with diabetic mice. Similarly, a 5.7-fold decrease in total leukocyte count was observed in the BAL fluid of vitamin D_3_-treated diabetic mice (Table 1).

## 4. Discussion

Alloxan (ALX) is an effective diabetes-inducing agent widely used in experimental animal models. ALX administration produces hyperglycemia, which leads to polyuria and weight loss [18]. In our study, blood glucose and body weight were measured 10 days after ALX administration. During the experimental period, diabetic mice exhibited lower body weight gain and hyperglycemia than nondiabetic mice. Both metabolic alterations may occur due to increased lipolysis and oxidative degradation of amino acids that increase tissue energy expenditure and body weight loss [19].

Few studies have demonstrated the effects of vitamin D_3_ supplementation on T1D in animal models. Nevertheless, results are controversial due to the use of different animal strains, protocols, doses, periods, and routes of supplementation. In our study, diabetic and nondiabetic mice supplemented with 800 IU (40000 IU/kg body weight/day) of vitamin D_3_ had higher serum 25(OH)_2_D levels than controls. According to Takiishi et al. (2014), this dose causes no significant alterations in serum P or Ca levels in nonobese diabetic (NOD) mice [14].

The role of vitamin D_3_ in body weight control is controversial. The presence of 1*α*–hydroxylase and VDR in adipose tissue cells suggests the involvement of vitamin D in body weight regulation [20]. Weight loss has been associated with Ca metabolism, which is regulated by Vitamin D_3_. The increase in Ca availability enhances oxidation, promotes apoptosis of adipocytes, and reduces the absorption of lipids due to the formation of insoluble molecules in the intestine [21]. However, Takiishi et al. found no difference in body weight variation between vitamin D_3_-treated NOD mice and controls [14]. Similarly, in our study, there was no significant difference in body weight variation between vitamin D_3_-treated mice and controls during the supplementation period.

The effects of vitamin D_3_ supplementation on glycemic control in animal models are controversial. Relative to controls, NOD mice treated with high doses of calcitriol (5 *µ*g/kg/alternate days) showed lower insulitis and a reduction in T cell counts [22]. However, vitamin D supplementation had little effect in reverting overt diabetes in streptozotocin-induced diabetic mice [23]. In the current study, vitamin D supplementation did not reduce blood glucose levels in diabetic mice compared to controls.

The major role of vitamin D_3_ in the body is to regulate the concentrations of Ca and P [8]. Few studies have focused on how vitamin D and its analogues may affect Ca homeostasis in T1D [24, 25]. In addition, diabetic individuals can exhibit low serum Ca and P levels due to renal disorders [26]. In the current study, serum concentrations of these ions were measured. Diabetic mice had lower serum Ca levels than controls and vitamin D_3_ supplementation increased these levels in the former. High 1,25(OH)_2_D levels stimulate bone turnover, renal excretion, and intestinal Ca absorption [7]. However, serum P levels in our study were significantly higher in vitamin D_3_-treated nondiabetic mice than controls. There was no significant difference in P levels between vitamin D_3_-treated and control diabetic mice.

Vitamin D is transported by albumin and vitamin D-binding protein in the bloodstream to the liver and kidneys, where it is activated [7, 8]. In the current study, liver enzymes (ALP, ALT, and AST) were measured to determine organ dysfunction in diabetic and nondiabetic mice. The concentrations of serum ALT, AST, and ALP were not significantly different between mice supplemented with vitamin D and controls. Measuring serum urea and creatinine levels is an alternative approach to estimate renal function [27]. In our study, diabetic mice showed low albumin levels and high serum levels of both creatinine and urea and a thickening of the Bowman's capsule compared to nondiabetic mice. These results may indicate renal failure [4]. However, vitamin D supplementation did not restore these renal parameters.

Alterations in hematological parameters are common in diabetes patients [28]. Hyperglycemia-induced oxidative stress in diabetic conditions leads to functional and morphological alterations of the erythrocyte membrane [29]. The increased nonenzymatic glycosylation of RBC membrane proteins and hyperglycemia could be responsible for the low Hb levels in these patients [30]. Hyperglycemia and oxidation of these proteins increase the production of lipid peroxides that lead to hemolysis of RBC [31]. In our study, diabetic mice had lower RBC counts and Hb and Htc levels than controls. Conversely, vitamin D_3_-treated diabetic mice showed RBC counts and Htc and Hb levels similar to those of nondiabetic mice. These findings suggest that vitamin D may affect erythropoiesis. Some studies have shown that VDR is expressed in the bone marrow by specific cell subsets such as stromal and accessory cells [32]. In addition, Aucella et al. reported that patients with chronic kidney disease undergoing hemodialysis showed a significant increase in Hb concentration and Htc level after four months of treatment with vitamin D [33].

Increased WBC counts are common in acute and chronic diabetes complications [34]. However Matough et al. observed lower WBC counts in diabetic mice as compared to nondiabetic animals [30]. In our study, diabetic mice showed lower mononuclear cell and WBC counts than control mice. Conversely, vitamin D_3_ treatment restored mononuclear cell and WBC counts on diabetic mice to levels similar to those of nondiabetic mice. Interestingly, vitamin D_3_-treated diabetic mice showed a reduction in pulmonary and peritoneal leukocyte counts compared to diabetic animals. Some studies have shown that VDR expression on leukocyte subsets [35] could increase leukocyte migration from tissues to peripheral blood.

Taken together, our results suggest that vitamin D supplementation may improve hematological parameters and reduce cell counts of BAL and PeL fluids during the course of diabetes.

## Figures and Tables

**Table 1:** Quantification of pulmonary and peritoneal leukocytes.

White cells (10^5^ cells/mL)						
Site	Group	*N*	Total	Macrophage	Lymphocyte	Neutrophil
Peritoneum	Control	9	11.2 ± 2.8	8.1 ± 2.3	2.95 ± 0.0	0.1 ± 0.0
	Vitamin D	7	16.0 ± 3.2	12.0 ± 2.2	3.4 ± 1.5	0.5 ± 0.1^**∗**^
	Diabetic	7	18.7 ± 3.0	14.8 ± 3.2	3.6 ± 0.8	0.2 ± 0.1
	Diabetic + vitamin D	7	8.8 ± 2.1^#^	7.6 ± 1.7	1.0 ± 0.5	0.3 ± 0.2
Lung	Control	3	4.6 ± 2.0	3.5 ± 1.9	0.9 ± 0.6	0.2 ± 0.1
	Vitamin D	4	9.1 ± 4.0	6.8 ± 2.4	2.0 ± 1.5	0.3 ± 0.2
	Diabetic	3	14.5 ± 4.0	11.5 ± 2.1	2.5 ± 1.4	0.9 ± 0.6
	Diabetic + vitamin D	3	2.1 ± 0.7^#^	19.9 ± 0.5	0.1 ± 0.1	0.1 ± 0.0

Mice were rendered diabetic by the injection of alloxan (60 mg/kg, i.v.), 10 days before vitamin D supplementation (800 IU/day, o.v.). BAL and PeL were performed 24 h thereafter. Values are expressed as means ± SD.

^**∗**^ *p* < 0.01 versus corresponding values in control group.

^#^ *p* < 0.05 versus corresponding values in diabetic group.
